# Cognitive Rehabilitation in Schizophrenia-Associated Cognitive Impairment: A Review

**DOI:** 10.3390/neurolint15010002

**Published:** 2022-12-29

**Authors:** Elli Zoupa, Olympia Bogiatzidou, Vasileios Siokas, Ioannis Liampas, Georgios Tzeferakos, Venetsanos Mavreas, Stelios Stylianidis, Efthimios Dardiotis

**Affiliations:** 1 Larisa Day Care Center of People with Alzheimer’s Disease, Association for Regional Development and Mental Health (EPAPSY), 15124 Marousi, Greece; 2 Department of Neurology, University Hospital of Larissa, Faculty of Medicine, School of Health Sciences, University of Thessaly, 41100 Larissa, Greece; 3 Department of Psychiatry, School of Medicine, University of Ioannina, 45110 Ioannina, Greece; 4 Department of Psychology, Panteion University of Social and Political Sciences, 14561 Athens, Greece

**Keywords:** cognition, schizophrenia, effectiveness, computerized remediation, cognitive training

## Abstract

Patients suffering from schizophrenia often experience cognitive disturbances. Cognitive rehabilitation—computerized or non-computerized—is widely known as an alternative way to enhance cognitive functioning in patients with schizophrenia. The aim of the present review was to examine the role of cognitive rehabilitation (both computerized and non-computerized) for the alleviation of cognitive impairment in schizophrenia patients. Fourteen relative studies were examined and included in the present review. The results revealed that both computerized and non-computerized cognitive rehabilitation could enhance cognitive functioning and more specifically memory, attention, executive functioning, processing speed and in a few cases, even non-cognitive impairments, such as other schizophrenia symptoms. The present results support the efficacy of cognitive rehabilitation in schizophrenia patients, regardless of whether it is computerized or non-computerized. As the randomized control trials (RCTs) are limited in number, there is urgent need for more RCTs and longitudinal studies combining different kinds of interventions, as well as systematic reviews and meta-analyses, in order to further investigate and confirm the current results.

## 1. Introduction

Schizophrenia is a serious mental disorder that affects up to 1% of the population worldwide, without significant differences in its prevalence among countries or between men and women [1,2,3]. In schizophrenia, the ability to perceive reality is disrupted, and the person experiences a set of psychiatric symptoms that negatively affect his/her mental and cognitive functions, psychosocial skills, and overall well-being [4]. Symptoms are classified as positive, including those that result from the exaggeration or distortion of the patient’s functions (delusions, hallucinations, bizarre behavior, thought disorder, inappropriate feelings), negative, including those resulting from the absence or flattening of normal functions (apathy, lethargy, hypersensitivity) [5] and disorganization, as well as cognitive deficits (dysfunctions regarding memory, processing speed, attention, and executive functions) [4].

Cognitive impairment is a core feature of schizophrenia. Patients with schizophrenia demonstrate cognitive deficits that vary between one and two standard deviations compared to healthy controls participants [6]. It is associated with both long-term disability and psychosocial impairments, apart from the cognitive decline itself, and it is estimated that 85% of patients suffer from cognitive disturbances [7]. Several studies reported that patients with schizophrenia exhibit cognitive disturbances, which interfere with their daily living and functioning, and it has been noted that cognitive impairment does not occur strictly due to psychotic symptoms [8]. Cognitive deficits can occur earlier than other manifestations and tend to be resistant to antipsychotic pharmacotherapy [9]. Although traditional antipsychotic medications have demonstrated some utility in treating the positive symptoms of the disease, current treatments are limited by their side effects and suboptimal efficacy in the management of negative symptoms [10]. The studies regarding second or atypical antipsychotics and their impact on cognition reported controversial findings. Some studies have shown beneficial effects [11,12], while others have mentioned the opposite [13,14].

Cognitive impairment predominantly includes processing and psychomotor speed, attention, memory (verbal and visual), working memory, executive functions, reasoning, visuospatial abilities, and metacognition [9,15]. Cognitive impairments are rather general and not restricted to one cognitive domain [16]. Of note, cognitive dysfunction can be present both during the acute phase of schizophrenia and during remission periods, deteriorating the overall functional impairment of affected individuals [9,15].

Cognitive deficits are considered as a complex interaction of genomic, neurobiological and neuroanatomic processes. For this reason, the focus is on the development of personalized treatments targeting neuroplasticity principals and combining pharmacotherapy with non-pharmacological treatments, such as Cognitive Rehabilitation Therapy [17]. The non-pharmacological interventions typically are time-limited [18]. CRT programs generally are distinguished as restorative and compensatory approaches, and they differ regarding the overall training settings (paper and pencil or computerized programs, group or individually implemented programs, in combination with other types of rehabilitation programs or not) [19].

Cognitive rehabilitation involves the implementation of behavioral interventions with an aim to improve and strengthen impaired cognitive functions (for example, memory, attention, executive functions, social cognition, metacognition). The main purpose of cognitive rehabilitation is to enable individuals to manage cognitive deficits more efficiently through multiple techniques and thus ameliorate their quality of life and activities of daily living [19,20].

To date, there is enough evidence to support the positive impact of cognitive rehabilitation on schizophrenia. It has been shown that the early application of rehabilitation schemes contributes decisively to slowing down the progression of symptoms and improving cognitive and mental functioning [19]. Cognitive rehabilitation programs, as mentioned above, can be implemented using paper and pencil or via computers and tablets. Their duration and complexity can be adjusted by the clinical expert to the specific needs of each individual. The whole process is based on the brain’s neuroplasticity dynamics and its ability to create new connections and modify neuronal circuits through systematic practice [19,20].

Regarding its efficacy, cognitive training, computerized or non-computerized, seems to present a slight to moderate impact on patients’ cognitive and daily functioning, quality of life, and well-being [21,22]. It is very important to add cognitive rehabilitation in a broader framework of holistic interventions, not only as monotherapies, in order to enhance everyday functioning and improve quality of life [23]. Relative reviews have mainly focused on the investigation of the effectiveness of a specific cognitive rehabilitation program, mostly via computerized cognitive training. Furthermore, most of these studies examined the effect of CRT, not exclusively on schizophrenia patients, but also on patients with other schizoaffective disorders.

The aim of the current review is to investigate the role and impact of cognitive rehabilitation (computerized or non-computerized and from various cognitive rehabilitations programs) on the cognitive dysfunction that occurs only in schizophrenia patients and consequently its effect on their psychosocial and daily functioning.

## 2. Materials and Methods

A non-systematic literature search was performed in PubMed in order to retrieve every relevant study published from 2000 until August 2021, when the last search was conducted. Every retrieved paper, as well as every relevant systematic review and meta-analysis, was manually scrutinized for relevant references. Articles were excluded according to the following criteria: irrelevant papers, articles not published in English, studies performed in animals, other study designs such as review and meta-analysis, retracted papers, and diagnosis other than schizophrenia (for example other schizoaffective disorders or psychosis). Additionally, regarding studies from the same research groups, we carefully checked and excluded those with overlapping data and selected those with the most relevant information.

We included observational trials, RCTs, and pilot and feasibility studies. The search queries combined the following key words: “schizophrenia”, “cognitive rehabilitation”, “computerized remediation”, “effectiveness”, and “cognitive”. All titles and abstracts retrieved were manually screened for eligibility. Full texts of the studies that qualified from the initial screening were reviewed in order to establish if an article fulfilled the inclusion criteria. Literature search and study selection were performed by 2 authors independently (E.Z., O.B.). A third author resolved potential discrepancies (E.D.). Eligible studies were involved in the qualitative analysis and, if appropriate, in the quantitative synthesis of the results. The reviewed studies are presented in a PRISMA flow diagram [24]. The procedure of literature search and selection with numbers of articles at each stage is presented in Figure S1. 

From each study, we extracted, when possible, the data presented in Table 1 (data extraction main characteristics of the included studies).

In addition, we extracted the studies’ settings and outcomes, as presented in Table 2.

## 3. Results

A literature search was performed on the PubMed online scientific database, and from the 38 screened studies, 14 met the inclusion criteria. In more detail, 15 studies were excluded since the patients were not diagnosed exclusively with schizophrenia, 1 study implemented a non-cognitive intervention program, and 8 studies were reviews or meta-analyses (Figure S1).

### 3.1. Cognitive Training Programs

In most studies included (*n* = 10) (Table 2), the cognitive rehabilitation programs were conducted through computerized cognitive programs. One of them was self-administered using a smartphone [25]. The cognitive functions that were mostly targeted were the following: attention and concentration, memory (working memory, verbal memory, visuospatial memory), executive functions, psychomotor speed, processing, learning, language, metacognition, problem solving, reasoning, and social cognition.

In the remaining studies (*n* = 4), the cognitive rehabilitation training was conducted with more traditional or alternative methods, such as paper and pencil exercises, role playing, social interaction, and enhancement of metacognitive thinking. In these studies, the cognitive functions that were mainly targeted were attention, memory, language, executive functions, and social cognition [21,26,27,28].

The total duration of the training programs of the reviewed articles spanned from 1 month to 4.5–5 months. A wide range of cognitive functions was trained through each of these cognitive training programs, targeted not only at improvements in each function exclusively, but also at generalization of these benefits in everyday living and functioning and alleviation of schizophrenia clinical outcomes and symptoms. The participants were schizophrenia inpatients or outpatients, most of them stable and/or on antipsychotic medication.

In the reviewed RCT studies, the antipsychotic medication was chlorpromazine equivalent doses. In more detail, half of the studies mentioned that the chlorpromazine equivalent doses consisted of atypical antipsychotics, while in the remaining studies there was not specific mention regarding their type. The CRT programs were implemented mostly for participants during their third decade of life; the patients’ ages at the onset of the disease was mostly during the early twenties; the duration of the disease was approximately 10 years, and the patients represented both sexes.

Since the above parameters were similar, it could be hypothesized that reported impacts in cognitive functioning did not occur as a result of these parameters.

### 3.2. Cognitive Improvements after Cognitive Rehabilitation

Cognitive improvement in various domains was observed in the reviewed studies (Table 2). Improvements in the following cognitive functions were reported with greater frequency: learning and memory (*n* = 6 studies); executive functions (mental flexibility, reasoning, working memory) (*n* = 6 studies)’ attention, concentration, and vigilance (*n* = 4 studies); processing speed (*n* = 3 studies); perception; and emotion perception and theory of mind (*n* = 1 studies) [18,19,22,25,28,29,30]. It should be mentioned that the aforementioned studies used various assessment tools regarding neuropsychological evaluation.

In more detail, Pena and colleagues (2018) combined cognitive training with social interventions and reported that combined interventions led to prolonged and more intensive beneficial effects in cognitive functioning [28]. Computerized cognitive training enhanced working memory, reasoning, attention, and general cognition in several studies in a period of 1.5–3 months [18,19,22,29,30].

Additionally, Dubreucq and colleagues (2019) offered psychosocial interventions, including psychoeducation, cognitive behavior therapy, cognitive remediation therapy, and social skills training, and reported moderate improvements in sustained attention, working memory, metacognition, and improvements in reactive mental flexibility [26].

Three studies conducted a follow-up assessment regarding cognitive improvement and reported that it remained stable and evident after 6 months [29], 1 year [26], and even after 5 years [23], raising questions about the factors that mediate these long-term effects; nevertheless, only one study was RCT [29].

### 3.3. Improvement in Non-Cognitive Outcomes after Cognitive Rehabilitation

Regarding the non-cognitive improvement that emerged after cognitive rehabilitation, beneficial effects in general psychopathology (negative and positive symptoms of schizophrenia), work quality, work habits, and global functioning were demonstrated (Table 2) [19,22,25,28], while it should be mentioned that the aforementioned studies used various assessment tools regarding neuropsychological evaluation.

Particularly, improvements in negative symptoms of schizophrenia [26,28], improvements in positive and negative symptoms of schizophrenia [21,22], and improvements in general clinical condition and schizophrenia symptoms [25] were reported. Furthermore, one study also detected a benefit in work quality and habits [19]. The review of the above studies highlighted the need for more holistic approaches and interventions, combining cognitive training, psychiatric medication, psychoeducation, and social skills’ enhancement in order to achieve non-cognitive improvements.

## 4. Discussion

As revealed by the included studies (Table 2), cognitive rehabilitation is capable of improving patients’ performance in specific cognitive domains, while this improvement was further shown in a few studies to be able to ‘transfer’ to a more general level of overall cognitive and daily functioning and to clinical parameters of schizophrenia symptoms. According to the reviewed studies, cognitive rehabilitation was able to enhance the majority of cognitive functions (attention/concentration and vigilance, learning, working memory, verbal and visual episodic memory, executive functions, logical thinking and reasoning, mental flexibility, processing speed, metacognition, language, and perception) mostly via computerized programs and also via paper and pencil tasks [18,19,22,25,28,29,30]. The aforementioned results agree with previous findings regarding cognitive rehabilitation and schizophrenia. Overall, relative meta-analyses have shown that cognitive rehabilitation leads to improved cognition, psychosocial and occupational function, and in some cases improvements in clinical symptoms of schizophrenia [20,31,32].

Neurocognitive domains, such as memory, reasoning, attention, processing speed, and executive functions are reported as possible beneficial mediators of psychosocial functioning and aid in alleviating schizophrenia symptoms’ disorganization [20,33,34]. This has been proven in various studies [19,21,22,25,26], while it has also been noted that cognitive rehabilitation therapy should not be a stand-alone therapy. Instead, it should be part of holistic programs aimed at cognition training and changes in the clinical condition of schizophrenia patients [26].

Most of the studies presented in this review used computerized intervention programs. The use of computerized interventions is considered as a beneficial factor for neural plasticity [35]. Computerized rehabilitation therapy provides multisensory stimulation, automatic adjustment of the difficulty level, and personalization of learning activities with structured, flexible, and standardized training tasks. Moreover, it could be more entertaining and induce the participants’ motivation, while also being relatively cost-effective [20].

Regarding the role of neuroplasticity on cognitive improvements, it was hypothesized that the beneficial effects of cognitive rehabilitation could stem from neuroplasticity reserves, enriched by cognitive training [36]. Additionally, a relevant study demonstrated changes in resting-state networks (for example of prefrontal, thalamic, and executive networks), anatomical connectivity (intra- and inter- hemispheric fibers), and perseveration of gray matter volumes after cognitive rehabilitation sessions [37]. Such results could reflect CRT’s effectiveness and induction of long-term changes, as evinced by the studies that showed perseverance of the benefits in the follow-up assessments [23,26,29]. In the reviewed articles, the beneficial effects of CRT were reported in participants during the third decade of their life.

It should be noted that there are some limitations in the present review. Firstly, we included articles with a relatively limited total number of participants and also studies lacking control groups. Moreover, few of the reviewed studies conducted a follow-up evaluation. The total duration and intensity of some cognitive rehabilitation programs was somewhat short and may be incapable of inducing cognitive functioning improvement, while pre- and post-implementation evaluation of the clinical symptoms of schizophrenia was not always performed. In addition, various assessment tools were used among studies regarding cognitive and non-cognitive evaluation of participants. Regarding the literature search, it was not systematic and conducted only at one online scientific database (PubMed), so the possibility that some eligible articles failed to be obtained cannot be completely excluded.

There are several issues that future studies could and need to address. Larger, randomized studies, preferably longitudinal, could better elucidate the possible long-term effects of cognitive training on schizophrenia patients. Fisher and colleagues (2010) reported that improvements in verbal learning/memory, processing speed, and global functioning were present 6 months after targeted cognitive training (auditory exercises, categorization tasks, and prediction exercises) [29]. Dubreucq and colleagues (2019) showed that combined cognitive rehabilitation therapy and psychosocial interventions led to moderate improvements in sustained attention and working memory and high improvements in reactive mental flexibility, negative symptoms of schizophrenia and metacognition [26]. In addition, another study demonstrated that neurocognitive exercises maintained improvements in cognition after 5 years of cognitive interventions [23]. More efforts like these are needed to evaluate the prolonged benefits of CRT in schizophrenia.

Moreover, the role of holistic interventions, since monotherapies do not seem to suffice in tackling the entirety of the disease burden, should be further investigated in future studies. Combining cognitive training, psychoeducation, social interventions, psychotherapy, and psychiatric interventions on cognition and functioning of schizophrenia patients could lead to long-term improvements [21,26,30].

In addition, rehabilitation programs for younger schizophrenia patients—where the effect of neuronal plasticity could be more intense—and the general impact of this intervention on neuroplasticity should be further investigated. Similarly, the potential neural mechanisms underlying the effects of cognitive rehabilitation therapies should be elucidated. Finally, it would be helpful to develop simple and/or self-administered cognitive training programs that could be easily used in a clinical setting, but in a community setting as well.

## 5. Conclusions

The current review suggests that cognitive rehabilitation therapies provide benefits in schizophrenia patients’ cognitive functioning, and in some cases, they even lead to improvements in their global functioning and clinical symptoms. These interventions—computerized or non-computerized—typically are low cost, easily administered, and overall cost-effective. These results could add knowledge and have an impact on long-term organizational decisions concerning the implementation, design, or remodeling of rehabilitative programs in terms of duration and treatments’ combination to achieve better and more durable cognitive and functioning beneficial effects. In conclusion, it is very important to design and implement neuropsychological rehabilitation programs that are focused not only on cognition per se, but on the enhancement of schizophrenia patients’ everyday living and quality of life as well.

## Figures and Tables

**Table 1:** Summary table of studies included in the present literature review—data extraction.

First Author	Country of Origin	Publication Year	Study Design	Set of Diagnostic Criteria	Number of Participants	Mean Age ± SD, Sex Distribution
Bor et al.	France	2011	Randomized—single-blind parallel—arms design (part of a larger RCT study)	Schizophrenia according to DSM-IV-TR criteria	*n* = 32 Cognitive Remediation therapy Group/patients: *n* = 8 Non-Cognitive Remediation Therapy Group/patients: *n* = 9 Healthy controls: *n* = 15	Cognitive Remediation therapy Group/patients: 30.5 (8.3), *n* = 6 males Non-Cognitive Remediation Therapy Group/patients: 28.5 (7.2), *n* = 6 males Healthy controls: 30.1 (7.5), *n* = 10 males
Nemoto et al.	Japan	2018	Feasibility study	Schizophrenia according to DSM-IV criteria	*n* = 22	32.1 (10.36) *n* = 10 males
Murthy et al.	USA and UK	2012	Open label multisite single sequence study	Schizophrenia according to DSM-IV-R criteria	*n* = 55	31.1 (7) *n* = 42 males
Buonocore et al.	Italy	2018	Monocentric retrospective study	Schizophrenia according to DSM-IV-TR criteria	*n* = 60 CRT/SRT: *n* = 27 CRT/SRT+: *n* = 33	CRT/SRT: *n* = 34.59 (10.27) CRT/SRT+: *n* = 35.20 (9.42) *n* = 35 males
Krzystanek et al.	Poland	2020	Multicenter, open-label randomized trial	Paranoid schizophrenia according to ICD-10 criteria	*n* = 290 Study group: 199 Reference group: 91	Study group: 32.0 (5.92), *n* = 114 males Reference group: 32.2 (6.94), *n* = 60 males
Lee	Korea	2013	RCT	Schizophrenia according to DSM-IV criteria	*n* = 60 Cog-trainer group: 30 Usual Rehabilitation group: 30	Cog-trainer group: 43.53 (4.87), *n* = 16 males Usual Rehabilitation group: 43.46 (3.53), *n* = 17 males
Mohammadi et al.	Iran	2014	Pre-experimental study (pre-test and post-test in a single group)	Schizophrenia according to DSM-IV-TR criteria	*n* = 15	Not specifically mentioned (Age: 18 or older)
Cellard et al.	Canada	2016	Feasibility study (case study design)	Schizophrenia according to DSM-IV criteria	*n* = 3	Case A:26 years old, male Case B: 26 years old, male Case C: 24 years old, male
Fisher et al.	USA	2010	RCT	Schizophrenia according to DSM-IV criteria	*n* = 32 Computer games group: *n* = 10 Control group: *n* = 12 Targeted Cognitive Training group: *n* = 10	Computer games group: 46.90 (9.17), *n* = 8 males Control group: 48.50 (6.94), *n* = 10 males Targeted Cognitive Training group: 42.90 (8.06), *n* = 7 males
Matsuda et al.	Japan	2016	RCT	Schizophrenia according to DSM-IV-TR criteria	*n* = 62 Cognitive Rehabilitation group: *n* = 31 Control group: *n* = 31	Cognitive Rehabilitation group: 36.39 (8.53), *n* = 17 males Control group: 37.77 (9.12), *n* = 18 males
Mak et al.	Poland	2013	RCT	Paranoid schizophrenia according to ICD-10 criteria	*n* = 81 Research group: *n* = 41 Control group: *n* = 40	Research group: 34 (11.07), *n* = 19 males Control group: 39 (12.99), *n* = 18 males
Dubreucq et al.	French	2019	FACE-SC-Longitudinal study	No specifically mentioned at this article	*n* = 183	33.91 (10.26) *n* = 144 males
Jaiswal et al.	India	2020	Quasi—experimental design (preliminary intervention study)	Paranoid schizophrenia according to ICD-10 criteria	*n* = 12 Intervention group: *n* = 6 Control group: *n* = 6	Intervention group: 31.33 (2.34), *n* = 6 males Control group: 28.50 (5.58), *n* = 6 males

CRT = Cognitive Remediation Therapy; DSM-TR = *Diagnostic and Statistical Manual of Mental Disorders*—text revision; FACE-SC = FundaMental Advanced Centers of Expertise—schizophrenia; *n* = number of observations; ICD = International Classification of Diseases; RCT = randomized control study; SD = standard deviation; SRT = standard rehabilitation therapy.

**Table 2:** Summary table—settings and outcomes of the included studies.

Author (Year)	Study Settings	Cognitive Rehabilitation Training	Training Intensity	Trained Modules	Significant Cognitive Improvements after Rehabilitation	Significant Non- Cognitive Improvements after Rehabilitation
Bor et al. (2011)	Outpatients (stable doses of antipsychotic medications for at least 3 months before the inclusion in the study)	RehaCom software	Total duration 7 weeks, 14 individual sessions (2 h length)	Attention/concentration, working memory, logical thinking, executive functions	Strong improvements in reasoning and attention (variations in cerebral functioning at fMRI)	Not mentioned
Nemoto et al. (2018)	Inpatients	Cognitive rehabilitation programs workbook style	Total duration 8 weeks, 20 min. per day	Exercises to improve cognitive function, interpersonal relationships, social context	Feasibility and accessibility of cognitive rehabilitation during the acute phase of schizophrenia	Improvements in global functioning
Murthy et al. (2011)	Outpatients (clinically stable in the previous 6 months and on regular antipsychotic medication)	BFP—computerized intervention	≥32 BFP training (≈40 sessions, at least 1 h, 5 times/week) sessions over 8–10 weeks	Speed and accuracy of auditory information processing	Significant effect on auditory processing speed	No significant effects on cognitive performance in general and functional capacity
Buonocore et al. (2018)	Outpatients (clinically stable in the last 3 months)	Computerized cognitive remediation therapy, performed with the Cogpack software and SRT	3 months of 3 1 h sessions a week and 6 months SRT and acute and 5-year follow-up assessment	Domain-specific neurocognitive exercises depending on patients’ impairments and graded difficulty	Cognitive abilities remained stable after 5 years in both groups (except of psychomotor speed and coordination)	Not mentioned
Krzystanek et al. (2020)	Outpatients (in symptomatic remission and stable schizophrenia symptoms at a mild level for at least 6 months prior study enrolment)	Self-administered cognitive training using a smartphone-based application (MONEO)	Cognitive training twice a week (study group) with increasing levels of difficulty and limited 3 cognitive trainings (reference group)	Not specifically mentioned, measurements of response time, rate of correct answers, rate of incorrect answers and rate of lack of reaction	Significant cognitive improvement in both the rate of correct answers and cognitive fatigability and slight improvement in the rate of incorrect answers	Significant improvement in the clinical condition, decreased schizophrenia symptoms
Lee (2013)	Inpatients (stable dose of antipsychotic therapy for at least 6 months)	Computerized cognitive training employed Cog-trainer software	Cog-trainer group: 20 sessions, 1 h, one or two times a week over 3 months UR group: 15 months duration	Cog-trainer group: attention and working memory UR group: social skills, vocational, recreational functioning, psychoeducation	Significant improvement in attention, concentration and working memory	Improvement in the work quality subscale and work habits of the work behavior inventory
Mohammadi et al. (2014)	Outpatients (clinically stable)	RehaCom software	20 individual sessions each 60 min, 2 sessions/week for 18 weeks	Attention/concentration, working memory and executive functions	Improvements in attention/vigilance, working memory and prospective and retrospective memory	No improvements in positive or negative symptoms of schizophrenia
Cellard et al. (2016)	Recruited from clinical settings without acute psychotic symptoms	Computerized cognitive remediation—CIRCuiTS	1 h sessions at least 3 days/week, for 40 sessions total	Attention, memory, executive functioning, and metacognitive skills	Improvements in verbal and visual episodic memory	Not specifically mentioned
Pena et al. (2018)	Not specifically mentioned (inpatients and outpatients probably from a previous RCT from which the sample was recruited)	REHACOP group: Cognitive rehabilitation based on paper and pencil tasks, role playing, active group discussions Control group: occupational group activities	4 months total duration, 90 min sessions, 3 days/week both groups	REHACOP group: attention, memory, language, executive functions, and social cognition control group: occupational activities	Improvements on processing speed and verbal memory, working memory, executive functions, emotion perception, theory of mind, social perception	Negative symptoms of schizophrenia
Fisher et al. (2010)	Not specifically mentioned (clinically stable patients recruited from mental health settings in the community)	TCT (software developed by Posit-Science, Inc.) and commercially available computerized games	TCT group: 12 participants completed 50 h training—10 participants completed 100 h training CG group: 5 days/week, 1 h/day (4–5 games/day)	TCT group: auditory exercises, visual system, categorization, prediction, association of information from auditory and visual stimuli CG group: 16 different games, e.g., visuospatial puzzle games, clue-gathering mystery games	Both TCT groups showed significant improvements in verbal learning/memory, cognitive control TCT-100 h training group showed improvements in processing speed and global condition	Not mentioned
Matsuda et al. (2016)	Outpatients	Original computer programme JCORES	Cognitive remediation group: 60 min., 2 sessions/week, 12 weeks total duration (plus 1/week metacognition enhancement and strategies teaching) control group: 12 weeks total; duration	attention, psychomotor speed, learning, memory, executive functions, verbal memory, verbal fluency, reasoning, problem solving, metacognition, strategies	Improvements on verbal memory and composite cognitive score	Improvements on general psychopathology on the positive and negative symptoms syndrome scale
Mak et al. (2013)	Not specifically mentioned (stable at remission period and pharmacological monotherapy)	RehaCom programme	16 sessions, twice/week (40 min.) 60 days total duration	Attention/concentration, topological memory	Moderate improvement in cognitive functioning (combined with pharmacological treatment and psychiatric rehabilitation)	Not mentioned
Dubreucq et al. (2019)	Not specifically mentioned—recruited from FACE-SZ cohort	CRT	At least 1 PI during the 1-year follow-up	Not specifically mentioned	Moderate improvement in sustained attention, working memory, high improvement in reactive mental flexibility (combined with PI intervention)	Moderate improvement in negative symptoms, mild effect on clinical global severity and on the level of insight into illness (combined with PI intervention)

BFP = brain fitness program; CG = cognitive condition; FACE-SC = FundaMental Advanced Centers of Expertise—schizophrenia; fMRI = functional magnetic resonance imaging; JCORES = Japanese Cognitive Rehabilitation Programme for Schizophrenia; PANSS = positive and negative syndrome scale; PI = psychosocial intervention; SRT = standard rehabilitation therapy; TCT = targeted cognitive training; UR = usual rehabilitation.

## Data Availability

Not applicable.

## Supplementary Materials

The following supporting information can be downloaded at: https://www.mdpi.com/article/10.3390/neurolint15010002/s1, Figure S1: PRISMA 2020 flowchart.
