# Supplementary figures and images for: Cognitive Rehabilitation in Schizophrenia-Associated Cognitive Impairment: A Review
Source: Neurol Int. 2022 Dec 29;15(1):12–23. doi: 10.3390/neurolint15010002 (PMC9844333; doi:10.3390/neurolint15010002)

Figure S1. PRISMA 2020 flowchart

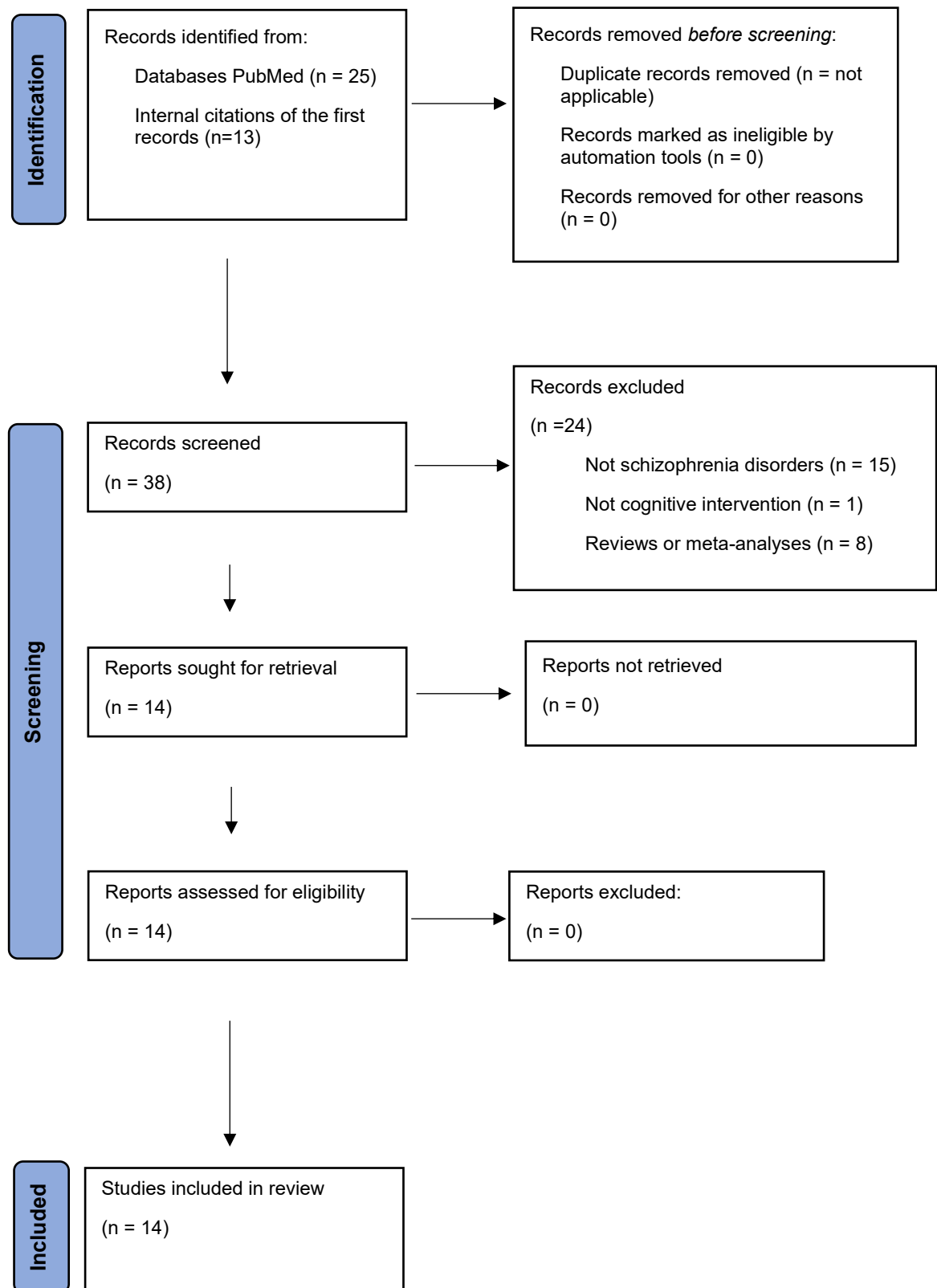

Supplement: Supplementary file 1 [file neurolint-15-00002-s001.zip › neurolint-2028193-supplementary.pdf]
